# Comprehensive Analysis of the Correlation Between Immune‐Inflammatory Biomarkers and Intestinal Necrosis in Patients With Acute Intestinal Ischemia

**DOI:** 10.1155/mi/8701105

**Published:** 2025-12-29

**Authors:** Yu Tian, Feifan Wang, Mingshuo Zhang, Rui Ding, Yimin Wang

**Affiliations:** ^1^ Department of Hand and Foot Surgery, First Hospital of Qinhuangdao, Qinhuangdao, 066000, China, qhdsdyyy.com; ^2^ Graduate School, Hebei North University, Zhangjiakou, 075000, China, hebeinu.edu.cn; ^3^ Graduate School, Hebei Medical University, Shijiazhuang, 050017, China, hebmu.edu.cn; ^4^ Department of General Surgery, First Hospital of Qinhuangdao, Qinhuangdao, 066000, China, qhdsdyyy.com

**Keywords:** acute intestinal ischemia, intestinal necrosis, neutrophil-to-lymphocyte ratio, relationship, risk factor, systemic immune-inflammation index

## Abstract

**Background:**

The relationship between immune‐inflammatory biomarkers, including neutrophil‐to‐lymphocyte ratio (NLR), platelet‐to‐lymphocyte ratio (PLR), and systemic immune‐inflammation index (SII) and intestinal necrosis in different types of acute intestinal ischemia remains unclear.

**Methods:**

A total of 138 patients with acute intestinal ischemia were divided into a nonnecrosis group (*n* = 65) and a necrosis group (*n* = 73). Least absolute shrinkage and selection operator (Lasso)‐logistic regression model was used to identify independent risk factors for intestinal necrosis. Multivariate logistic regression was conducted to assess the associations between NLR and SII levels and intestinal necrosis. Sensitivity analyses were performed using smooth curve fitting, threshold effect analysis, subgroup analysis, and propensity score matching (PSM).

**Results:**

The optimal thresholds of NLR and SII for differentiating intestinal necrosis were 8.85 and 1492.11, respectively, while PLR showed no significant association. In Lasso‐logistic regression analysis, vascular intestinal ischemia, NLR ≥ 8.85, SII≥ 1492.11, C‐reactive protein (CRP), and D‐dimer > 0.5 mg/L were independent risk factors for intestinal necrosis. After adjusting for potential confounding variables, the multivariate logistic regression analysis showed a positive correlation between natural logarithm (Ln)‐NLR (OR = 20.187, 95% CI = 3.788–107.578, *p*  < 0.001) and Ln‐SII (OR = 5.375, 95% CI = 1.141–25.313, *p*  < 0.033) and intestinal necrosis. Subgroup analysis showed a significant interaction between NLR, intestinal necrosis, and coronary heart disease (*p* interaction = 0.016). Smooth curve fitting indicated the relationship between Ln‐NLR and Ln‐SII and intestinal necrosis was nonlinear, with inflection points at 1.78 (NLR = 5.93) and 7.32 (SII = 1510.20), respectively. When NLR > 5.93 or SII < 1510.20, the risk of intestinal necrosis significantly increased. The associations remained robust after PSM.

**Conclusion:**

This study demonstrates that elevated levels of NLR and SII are significantly associated with intestinal necrosis, suggesting that these biomarkers may help identify patients at higher risk of intestinal necrosis in acute intestinal ischemia. However, prospective studies are needed to validate their predictive value.

## 1. Introduction

Acute intestinal ischemia is a gastrointestinal emergency characterized by a lack of mesenteric blood supply or obstruction of blood return due to various etiologies and risk factors, leading to varying degrees of intestinal wall lesions and damage [1]. It can be categorized into two major types: mechanical causes outside the blood vessels, such as intestinal adhesions, torsion, strangulated obstruction, or incarcerated hernias, and ischemic intestinal diseases caused by vascular or intravascular factors, including arterial or venous ischemia, with arterial ischemia being more prevalent [1–3].

Although the incidence of acute intestinal ischemia is relatively low, it can result in transmural necrosis, leading to intestinal perforation, sepsis, and even death. Without prompt treatment, the mortality rate can reach 60%–80% [4, 5]. Early recognition of intestinal necrosis is the key determinant of prognosis. However, clinical manifestations such as abdominal pain, nausea, vomiting, and bloody stools are nonspecific, and laboratory findings lack sufficient sensitivity and specificity [6–8]. Imaging techniques, particularly computed tomography (CT) and CT angiography (CTA), have markedly improved diagnostic accuracy for intestinal ischemia [9]. Yet, even with advanced imaging, detecting intestinal necrosis at an early stage remains challenging—often diagnosis occurs only after irreversible transmural necrosis has developed, when surgical outcomes are poor and complications such as short bowel syndrome may ensue [10–12]. This highlights the urgent need for reliable early biomarkers to predict intestinal necrosis before radiologic or clinical deterioration occurs.

Several serological biomarkers have been investigated to aid early diagnosis, such as serum lactate, D‐dimer, C‐reactive protein (CRP), and procalcitonin. Elevated lactate levels may reflect tissue hypoperfusion but are nonspecific and often rise only after advanced ischemia [13]. D‐dimer has shown moderate sensitivity but poor specificity, as it can also increase in sepsis, malignancy, or thrombosis [14]. Similarly, CRP and procalcitonin can suggest systemic inflammation or infection but cannot reliably distinguish reversible ischemia from irreversible necrosis [15, 16]. These limitations underscore the urgent need for novel, accessible, and more accurate biomarkers to predict intestinal necrosis at an early stage.

In this context, inflammation‐based hematological indices derived from routine blood tests, such as the neutrophil‐to‐lymphocyte ratio (NLR), platelet‐to‐lymphocyte ratio (PLR), and systemic immune‐inflammation index (SII) have gained increasing attention. Emerging evidence indicates that intestinal ischemia and necrosis disrupt the mucosal barrier, causing bacterial translocation and systemic inflammatory activation [17]. NLR and PLR are markers that reflect systemic inflammation and have been shown to predict outcomes in various conditions, including malignancies, chronic obstructive pulmonary disease, and sepsis [18–20]. Elevated NLR and PLR levels have been linked to intestinal necrosis in certain pathological conditions [21, 22]. However, studies investigating their association with intestinal necrosis are limited and mainly focused on specific subtypes of ischemia. The SII, a novel biomarker reflecting the balance between inflammation and immune status [23], has been associated with various diseases, including malignancies, myocardial infarction, and stroke [24–27]. However, its role in acute intestinal ischemia and intestinal necrosis remains unexplored.

Therefore, this study aimed to investigate the risk factors for intestinal necrosis in patients with different types of acute intestinal ischemia and to evaluate the relationship between NLR, PLR, and SII, and intestinal necrosis, providing potential early biomarkers to guide clinical decision‐making.

## 2. Materials and Methods

### 2.1. Population Selection

Clinical data from 138 patients with acute intestinal ischemia admitted to the Department of General Surgery at the First Hospital of Qinhuangdao from January 1, 2021, to December 31, 2024, were collected through the electronic medical record system. This study was approved by the Ethics Committee of the First Hospital of Qinhuangdao (No. 2025 K‐157‐01). The inclusion criteria were as follows: (1) patients suspected of acute intestinal ischemia based on abdominal symptoms, signs, and imaging studies (e.g., CTA, enhanced CT); (2) patients who underwent emergency exploratory laparotomy at our hospital; (3) patients with complete clinical records. The exclusion criteria were as follows: (1) patients with intestinal ischemia caused by malignant tumors; (2) patients with clinically suspected of bowel ischemia but did not undergo laparotomy; (3) patients with a history of radiation or chemotherapy; (4) patients with mental illnesses, cognitive dysfunction, or other conditions that prevent them from cooperating with treatment; (5) patients with incomplete medical records.

### 2.2. Information Collection

Basic patient information, including age, gender, body mass index (BMI), body temperature, underlying comorbidities, cause of illness, and time of onset, was collected through the electronic medical record system. Cause of illness can be categorized into two major types: mechanical causes outside the blood vessels, such as intestinal adhesions, torsion, strangulated obstruction, or incarcerated hernias, and ischemic intestinal diseases caused by vascular factors, including mesenteric artery embolism, arterial or venous thrombosis, and nonocclusive causes. Clinical laboratory indicators, including white blood cell count (WBC), neutrophil count, lymphocyte count, platelet count (PLT), hemoglobin (HGB), blood potassium, blood sodium, blood glucose, albumin, serum creatinine (Scr), lactate dehydrogenase (LDH), CRP, and D‐dimer levels, were also collected on admission to the hospital. NLR was calculated as neutrophil count/lymphocyte count, PLR as PLT/lymphocyte count, and SII as PLT × neutrophil count/lymphocyte count. Pathological examination of resected specimens from patients who underwent intestinal resection was performed to confirm the diagnosis of intestinal ischemia and necrosis. All specimens were pathologically examined to confirm full thickness small bowel wall necrosis. Categorical variables such as blood potassium, blood sodium, and D‐dimer were converted based on previous studies [28–30].

### 2.3. Covariates Selection

Covariates included in our study that may affect the association between NLR, SII, and intestinal necrosis include age, gender, BMI, hypertension, diabetes, coronary heart disease, and clinical variables that were significantly different between the nonnecrosis and necrosis groups.

### 2.4. Statistical Analysis

Data analysis was performed using SPSS (Version 25.0), R Studio (Version 4.2.0), and EmpowerStats (Version 5.0). Continuous variables were tested for normality using the Shapiro–Wilk test. Normally distributed variables are presented as mean ± standard deviation and compared using the independent samples *t*‐test, while nonnormally distributed variables are presented as median (interquartile range) and compared using the Mann–Whitney *U* test. Additionally, we compared categorical variables between different groups using the chi‐square test. To prevent model overfitting and identify the most informative predictors of intestinal necrosis, all clinical variables were entered into a least absolute shrinkage and selection operator (Lasso)‐logistic regression model. The optimal penalty parameter (*λ*) was determined using 10‐fold cross‐validation based on the minimum mean cross‐validated error criterion. Variables with nonzero coefficients in the optimal *λ* model were retained for subsequent multivariate logistic regression analysis. Receiver operating characteristic (ROC) curves were established to evaluate the predictive ability of variables for intestinal necrosis. We observed that the distribution of NLR and SII data was uneven and significantly skewed (Supporting Information: Figure S1). We performed a natural logarithm (Ln) transformation on NLR and SII (Ln‐NLR and Ln‐SII) to make them as close to a normal distribution as possible (Supporting Information: Figure S1). Based on the quartiles of Ln‐NLR and Ln‐SII, they were converted into four‐category variables and grouped for analysis. To further explore the association between Ln‐NLR, Ln‐SII, and intestinal necrosis, a multivariate logistic regression model was used. Three models were applied: Model 1: no adjustment for other variables; Model 2: adjustment for age, gender, BMI, and comorbidities; Model 3: adjustment for age, gender, BMI, comorbidities, and clinical variables that differed between the nonnecrosis and necrosis groups. Moreover, multicollinearity among the variables was considered by evaluating variance inflation factors (VIF) [31]. A VIF value > 5 was considered indicative of significant collinearity. Additionally, threshold effect analysis was performed using a generalized additive model (GAM) with restricted cubic splines (3 knots) to explore potential nonlinear relationships between Ln‐NLR, Ln‐SII, and intestinal necrosis. To reduce the risk of overfitting given the limited sample size, 10‐fold cross‐validation was applied to verify model stability. Subgroup analysis and interaction analysis were performed to assess the heterogeneity of the association between NLR, SII, and intestinal necrosis in different subgroups. Finally, propensity score matching (PSM) analysis was used to control for confounding factors and verify the stability of the association between NLR, SII, and intestinal necrosis. Furthermore, the statistical power was calculated by using the PS Software [32]. In our cohort of 138 patients, according to the optimal cutoff values, 77 (55.8%) were classified into the high‐NLR group and 81 (58.7%) into the high‐SII group. Assuming a two‐sided significance level of *α* = 0.05 and a statistical power of 0.80, we would be able to detect true odds ratios (ORs) of ≥2.77 for NLR and ≥2.68 for SII, respectively. All statistical tests were two‐sided, and a *p*‐value <0.05 was statistically significant.

## 3. Results

### 3.1. Clinical Characteristics of Acute Intestinal Ischemia Patients

According to the inclusion and exclusion criteria, clinical data from 138 patients with acute intestinal ischemia were collected. The median age of the patients was 68.5 years, with 94 males and 44 females. The median NLR was 10.05, the median PLR was 229.68, and the median SII was 1947.98. In terms of the cause of illness, the incidence of nonvascular intestinal ischemia was significantly higher than that of vascular intestinal ischemia (80.44% vs. 19.56%). Additionally, the majority (63.77%) of patients had an onset time of more than 12 h.

Based on whether intestinal necrosis occurred, the patients were divided into a nonnecrosis group (*n* = 65) and a necrosis group (*n* = 73). The results showed that the NLR (14.60 [9.55, 20.69] vs. 6.43 [3.27, 9.94], *p*  < 0.001) and SII (2858.79 [1558.92, 4598.18] vs. 1112.14 [699.77, 1971.34], *p*  < 0.001) levels were higher in intestinal necrosis patients. However, there was no significant difference in PLR levels between the two groups (*p*  > 0.05) (Table 1). Additionally, the cause of illness in the necrosis group was more likely to be vascular (*p*  < 0.001), and the onset time was longer (*p* = 0.003) (Table 1). In patients with intestinal necrosis, the WBC (*p* = 0.034), blood glucose (*p* = 0.040), Scr (*p*  < 0.001), LDH (*p*  < 0.001), CRP (*p*  < 0.001), and D‐dimer (*p*  < 0.001) levels were higher, the incidence of hyponatremia was higher (*p* = 0.032), and albumin levels were lower (*p* = 0.006) (Table 1). There were no differences in age, gender, hypertension, diabetes, coronary heart disease, BMI, body temperature, HGB, PLT, blood potassium, and other clinical characteristics between the two groups (*p*  > 0.05) (Table 1).

**Table 1:** Characteristics of the patients with acute intestinal ischemia.

Characteristic	Total patient (*n* = 138)	Nonnecrosis group (*n* = 65)	Necrosis group (*n* = 73)	*p*
Age	68.50 (58.00, 76.75)	68.00 (58.00, 76.00)	69.00 (60.00, 77.00)	0.813
Gender	—	—	—	0.641
Male	94 (68.12%)	43 (66.15%)	51 (69.86%)	—
Female	44 (31.88%)	22 (33.85%)	22 (30.14%)	—
Hypertension	—	—	—	0.054
No	102 (73.91%)	53 (81.54%)	49 (67.12%)	—
Yes	36 (26.09%)	12 (18.46%)	24 (32.88%)	—
Diabetes	—	—	—	0.997
No	121 (87.68%)	57 (87.69%)	64 (87.67%)	—
Yes	17 (12.32%)	8 (12.31%)	9 (12.33%)	—
Coronary heart disease	—	—	—	0.866
No	116 (84.06%)	55 (84.62%)	61 (83.56%)	—
Yes	22 (15.94%)	10 (15.38%)	12 (16.44%)	—
Etiology	—	—	—	**<0.001**
Nonvascular	111 (80.44%)	61 (93.85%)	50 (68.49%)	—
Vascular	27 (19.56%)	4 (6.15%)	23 (31.51%)	—
Onset time (h)	—	—	—	**0.003**
≤12	50 (36.23%)	32 (49.23%)	18 (24.66%)	—
>12	88 (63.77%)	33 (50.77%)	55 (75.34%)	—
BMI (kg (m)^2^)	22.10 (19.65, 25.55)	22.05 (19.35, 25.40)	22.50 (20.30, 25.75)	0.688
Temperature (°C)	36.50 (36.30, 36.70)	36.50 (36.20, 36.60)	36.50 (36.30, 36.80)	0.205
WBC (10^9^/L)	9.16 (6.10, 11.90)	8.99 (5.94, 10.90)	10.25 (6.73, 12.98)	**0.034**
NLR	10.05 (6.30, 17.05)	6.43 (3.27, 9.94)	14.60 (9.55, 20.69)	**<0.001**
PLR	229.68 (148.56, 363.26)	229.27 (139.62, 318.97)	247.67 (163.01, 419.64)	0.095
SII ( ^∗^10^9^)	1947.98 (1040.93, 3347.43)	1112.14 (699.77, 1971.34)	2858.79 (1558.92, 4598.18)	**<0.001**
HGB (g/L)	126.94 ± 27.72	130.83 ± 24.86	123.48 ± 29.78	0.120
PLT (10^9^/L)	206.50 (151.50, 259.50)	193.00 (167.00, 243.00)	213.00 (144.00, 280.00)	0.395
Potassium (mmol/L)	—	—	—	0.304
3.5–5.5	106 (76.81%)	53 (81.54%)	53 (72.60%)	—
<3.5	26 (18.84%)	11 (16.92%)	15 (20.55%)	—
> 5.5	6 (4.35%)	1 (1.54%)	5 (6.85%)	—
Sodium (mmol/L)	—	—	—	**0.032**
≥135	118 (85.51%)	60 (92.31%)	58 (79.45%)	—
<135	20 (14.49%)	5 (7.69%)	15 (20.55%)	—
Glucose (mmol/L)	7.56 (6.32, 10.62)	7.11 (6.11, 8.86)	8.14 (6.69, 11.06)	**0.040**
Albumin (g/L)	34.85 (27.33, 41.73)	37.30 (30.60, 43.50)	31.40 (25.50, 39.60)	**0.006**
Scr (*μ*mol/L)	74.15 (56.23, 124.20)	65.90 (53.70, 77.90)	90.50 (63.50, 162.80)	**<0.001**
LDH (U/L)	217.50 (185.00, 301.25)	198.00 (166.00, 238.00)	261.00 (201.00, 349.00)	**<0.001**
CRP (mg/L)	41.99 (9.93, 126.65)	12.50 (1.38, 40.97)	108.70 (40.43, 197.30)	**<0.001**
D‐dimer (mg/L)	—	—	—	**<0.001**
≤0.5	20 (14.49%)	17 (26.15%)	3 (4.11%)	—
> 0.5	118 (85.51%)	48 (73.85%)	70 (95.89%)	—

*Note:* *p* < 0.05 is indicated in bold.

### 3.2. Predictive Factors for Intestinal Necrosis in Acute Intestinal Ischemia Patients

**Figure 1:**
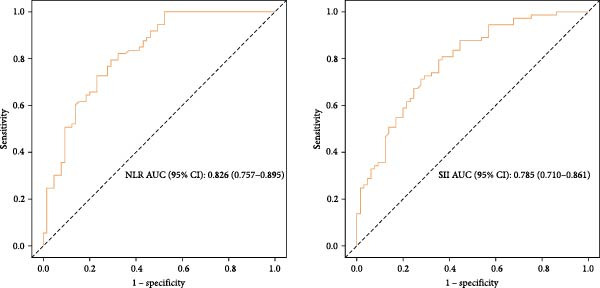
ROC curve of NLR and SII for predicting intestinal necrosis in patients with acute intestinal ischemia.

**Figure 3:**
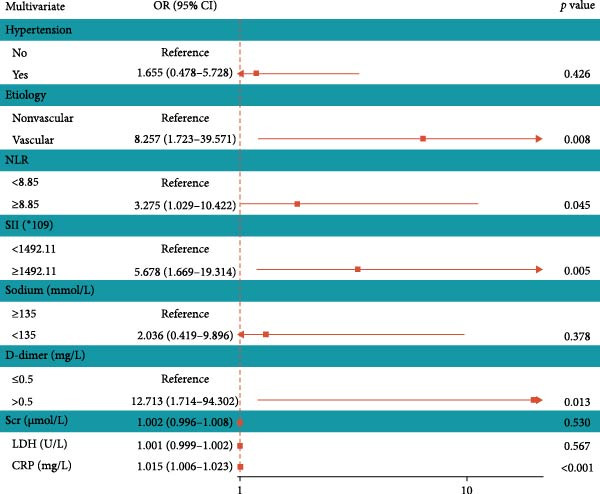
Multivariable logistic regression analysis investigating factors associated with intestinal necrosis in acute intestinal ischemia patients.

To explore the optimal cutoff values for NLR and SII in predicting intestinal necrosis in acute intestinal ischemia patients, ROC curves were constructed (Figure 1). The ROC curve showed that the optimal cutoff value for NLR in predicting intestinal necrosis was 8.85, with an AUC of 0.826 (95% CI: 0.757–0.895), a sensitivity of 0.708 (95% CI: 0.597–0.818), and a specificity of 0.795 (95% CI: 0.702–0.887). The optimal cutoff value for SII in predicting intestinal necrosis was 1492.11, with an AUC of 0.785 (95% CI: 0.710–0.861), a sensitivity of 0.646 (95% CI: 0.530–0.762), and a specificity of 0.795 (95% CI: 0.702–0.887) (Table 2). Based on the optimal cutoff values, NLR and SII were converted into binary variables. Subsequently, we explored the risk factors for intestinal necrosis in acute intestinal ischemia patients. First, all baseline demographic, clinical, and laboratory variables were included as candidate predictors in the Lasso regression model. The coefficient profiles of these variables are displayed in Figure 2A. Using a 10‐fold cross‐validation procedure to determine the optimal regularization parameter, the model achieved the minimum mean cross‐validated error at *λ* = 0.033 (Figure 2B). At this *λ* value, nine variables with nonzero coefficients were retained, including hypertension, cause of illness, NLR, SII, blood sodium, Scr, LDH, CRP, and D‐dimer. Based on these screened variables, a multivariate logistic regression model was further established. The results showed that vascular intestinal ischemia (OR = 8.257, 95% CI = 1.723–39.571, *p* = 0.008), NLR ≥ 8.85 (OR = 3.275, 95% CI = 1.029–10.422, *p* = 0.045), SII ≥ 1492.11 (OR = 5.678, 95% CI = 1.669–19.314, *p* = 0.005), CRP (OR = 1.015, 95% CI = 1.006–1.023, *p*  < 0.001), and D‐dimer > 0.5 mg/L (OR = 12.713, 95% CI = 1.714–94.302, *p* = 0.013) were independent risk factors for intestinal necrosis in acute intestinal ischemia patients (Figure 3).

Figure 2Variable selection using Lasso regression. (A) The variation characteristics of the coefficient of clinical variables. (B) The selection process of the optimum value of the parameter *λ* in the Lasso regression model by 10‐fold cross‐validation method.

**Figure:**
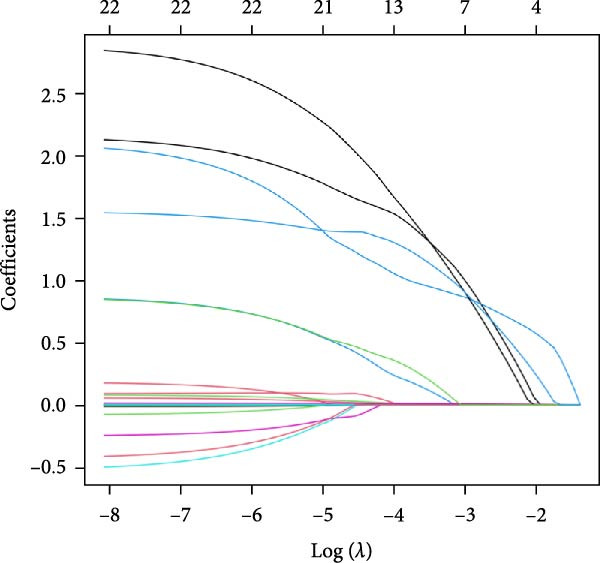
(A)

**Figure:**
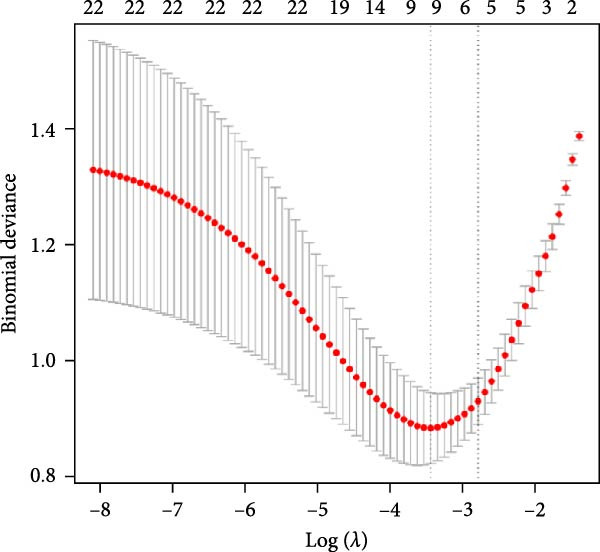
(B)

**Table 2:** The value of NLR and SII for predicting intestinal necrosis in patients with acute intestinal ischemia.

Variable	AUC (95% CI)	Sensitivity (95% CI)	Specificity (95% CI)	Optimal cutoff
NLR	0.826 (0.757–0.895)	0.708 (0.597–0.818)	0.795 (0.702–0.887)	8.85
SII	0.785 (0.710–0.861)	0.646 (0.530–0.762)	0.795 (0.702–0.887)	1492.11

### 3.3. Association Between NLR, SII, and Intestinal Necrosis in Acute Intestinal Ischemia Patients

In the statistical analysis, we observed that the distribution of NLR and SII data was uneven and significantly skewed. To accurately assess, whether there is a dose‐response relationship between NLR, SII, and intestinal necrosis, we performed a Ln transformation on NLR and SII (Ln‐NLR and Ln‐SII) to make them as close to a normal distribution as possible. Supporting Information: Table S1 shows the clinical characteristics of patients grouped by Ln‐NLR quartiles. The results showed that as the Ln‐NLR level increased, the levels of WBC, PLR, SII, Scr, LDH, and CRP also increased (*p*  < 0.05). Compared with the Ln‐NLR Q1 group, the incidence of intestinal necrosis was higher in the Ln‐NLR Q2, Q3, and Q4 groups (*p*  < 0.001) (Supporting Information: Table S1). Supporting Information: Table S2 shows the clinical characteristics of patients grouped by Ln‐SII quartiles. The results showed that as the Ln‐SII level increased, the levels of WBC, PLT, NLR, PLR, blood glucose, and LDH also increased (*p*  < 0.05). Compared with the Ln‐SII Q1 group, the incidence of intestinal necrosis was higher in the Ln‐SII Q2, Q3, and Q4 groups (*p*  < 0.001) (Supporting Information: Table S2).

To further study the correlation between Ln‐NLR, Ln‐SII, and intestinal necrosis, we constructed three multivariate logistic regression models. In Model 1, multivariate logistic regression analysis showed that Ln‐NLR (OR = 9.820, 95% CI = 4.402–21.905, *p*  < 0.001) and Ln‐SII (OR = 4.303, 95% CI = 2.453–7.551, *p*  < 0.001) were significantly positively correlated with intestinal necrosis. In Model 3, which adjusted for all covariates, the levels of Ln‐NLR and Ln‐SII in patients were still significantly associated with intestinal necrosis (Ln‐NLR: OR = 20.187, 95% CI = 3.788–107.578, *p*  < 0.001; Ln‐SII: OR = 5.375, 95% CI = 1.141–25.313, *p* = 0.033), meaning that for every 1‐unit increase in Ln‐NLR and Ln‐SII levels, the risk of intestinal necrosis increased by 19.187 times and 4.375 times, respectively (Table 3). We further transformed the Ln‐NLR and Ln‐SII from continuous variables into categorical variables (quartiles) for sensitivity analysis. The results showed that for Ln‐NLR and Ln‐SII, in Models 1 and 2, compared with the Q1 group, the risk of intestinal necrosis was higher in the Q2 (Models 1: Ln‐NLR: OR = 6.889, 95% CI = 1.993–23.807, *p* = 0.002; Ln‐SII: OR = 3.158, 95% CI = 1.084–9.204, *p* = 0.035; Models 2: Ln‐NLR: OR = 8.425, 95% CI = 2.233–31.785, *p* = 0.002; Ln‐SII: OR = 3.869, 95% CI = 1.271–11.783, *p* = 0.017), Q3 (Models 1: Ln‐NLR: OR = 18.600, 95% CI = 5.192–66.638, *p*  < 0.001; Ln‐SII: OR = 9.600, 95% CI = 3.167–29.105, *p*  < 0.001; Models 2: Ln‐NLR: OR = 25.210, 95% CI = 6.250–101.691, *p*  < 0.001; Ln‐SII: OR = 12.770, 95% CI = 3.762–43.350, *p*  < 0.001), and Q4 (Models 1: Ln‐NLR: OR = 37.458, 95% CI = 9.588–146.340, *p*  < 0.001; Ln‐SII: OR = 13.500, 95% CI = 4.301–42.375, *p*  < 0.001; Models 2: Ln‐NLR: OR = 44.641, 95% CI = 10.404–191.542, *p*  < 0.001; Ln‐SII: OR = 15.383, 95% CI = 4.626–51.157, *p*  < 0.001) groups, and there was also a significant dose‐response relationship between Ln‐NLR, Ln‐SII, and intestinal necrosis (*p*‐trend < 0.001). In Model 3, the association between Ln‐NLR, Ln‐SII, and intestinal necrosis still existed. Compared with the Q1 group, the risk of intestinal necrosis in the Ln‐NLR Q4 group increased by 29.172 times (OR = 30.172, 95% CI = 1.687–539.644, *p* = 0.021), and there was still a significant dose‐response relationship (*p*‐trend = 0.005). Compared with the Q1 group, the risk of intestinal necrosis in the Ln‐SII Q4 groups increased (OR > 1), but the difference was not statistically significant (*p* > 0.05) (Table 3). In this study, all variables included in the multivariate logistic regression model had VIFs < 5, suggesting that multicollinearity was not a concern (Supporting Information: Table S3). Therefore, the multivariate logistic regression model was considered statistically stable.

**Table 3:** Multivariable logistic regression models for the association between Ln‐NLR, Ln‐SII, and intestinal necrosis in acute intestinal ischemia patients.

Variable	Model 1			Model 2			Model 3	
	OR (95% CI)	*p*		OR (95% CI)	*p*		OR (95% CI)	*p*
Ln‐NLR								
Continuous	9.820 (4.402–21.905)	**<0.001**		11.258 (4.710–26.912)	**<0.001**		20.187 (3.788–107.578)	**<0.001**
Quartiles								
Q1	1.000 (Reference)	—		1.000 (Reference)	—		1.000 (Reference)	—
Q2	6.889 (1.993–23.807)	**0.002**		8.425 (2.233–31.785)	**0.002**		12.163 (1.478–100.099)	**0.020**
Q3	18.600 (5.192–66.638)	**<0.001**		25.210 (6.250–101.691)	**<0.001**		36.978 (3.354–407.690)	**0.003**
Q4	37.458 (9.588–146.340)	**<0.001**		44.641 (10.404–191.542)	**<0.001**		30.172 (1.687–539.644)	**0.021**
*p*‐trend		**<0.001**			**<0.001**			**0.005**
Ln‐SII								
Continuous	4.303 (2.453–7.551)	**<0.001**		4.469 (2.474–8.075)	**<0.001**		5.375 (1.141–25.313)	**0.033**
Quartiles								
Q1	1.000 (Reference)	—		1.000 (Reference)	—		1.000 (Reference)	—
Q2	3.158 (1.084–9.204)	**0.035**		3.869 (1.271–11.783)	**0.017**		7.857 (1.153–53.525)	**0.035**
Q3	9.600 (3.167–29.105)	**<0.001**		12.770 (3.762–43.350)	**<0.001**		5.605 (0.620–50.665)	0.125
Q4	13.500 (4.301–42.375)	**<0.001**		15.383 (4.626–51.157)	**<0.001**		6.031 (0.334–108.875)	0.223
*p*‐trend		**<0.001**			**<0.001**			0.246

*Note:* Model 1: no covariates were adjusted. Model 2: age, gender, BMI, hypertension, diabetes, and coronary heart disease were adjusted. Model 3: age, gender, BMI, hypertension, diabetes, coronary heart disease, etiology, onset time, WBC, NLR, SII, sodium, glucose, albumin, Scr, LDH, CRP, and D‐dimer were adjusted. *p* < 0.05 is indicated in bold.

### 3.4. The Nonlinear Relationship Between NLR, SII, and Intestinal Necrosis in Acute Intestinal Ischemia Patients

We used smooth curve fitting based on a generalized additive model with restricted cubic splines (3 knots) for Model 3 to visually describe the correlation between Ln‐NLR, Ln‐SII, and intestinal necrosis. To minimize potential overfitting due to the limited sample size, 10‐fold cross‐validation was applied to internally validate the model stability. The results showed that there was a nonlinear relationship between Ln‐NLR, Ln‐SII, and intestinal necrosis (Figure 4). Subsequently, we performed a threshold effect analysis to further clarify their relationship. The inflection point for Ln‐NLR was determined as 1.78 (NLR = 5.93) (log‐likelihood ratio *p* = 0.006), indicating that when Ln‐NLR is less than 1.78, there is no correlation between Ln‐NLR and intestinal necrosis. However, when Ln‐NLR is greater than 1.78, for every one‐unit increase in Ln‐NLR, the risk of intestinal necrosis increases by 4.63 times (OR = 5.634, 95% CI = 1.503–21.126, *p* = 0.010). For Ln‐SII, the inflection point was 7.32 (SII = 1510.20) (log‐likelihood ratio *p* = 0.005), and when Ln‐SII is less than 7.32, for every one‐unit increase in Ln‐SII, the risk of intestinal necrosis increases by 37.57 times (OR = 38.565, 95% CI = 3.032–490.497, *p* = 0.005). However, when Ln‐SII is greater than 7.32, the correlation between Ln‐SII and intestinal necrosis disappeared, suggesting that further increases in Ln‐SII did not significantly elevate the risk of intestinal necrosis (Table 4).

Figure 4The nonlinear associations between the Ln‐NLR (A), Ln‐SII (B), and intestinal necrosis in acute intestinal ischemia patients. The solid red line represents the smooth curve fit between variables. Blue bands represent the 95% confidence interval from the fit.

**Figure:**
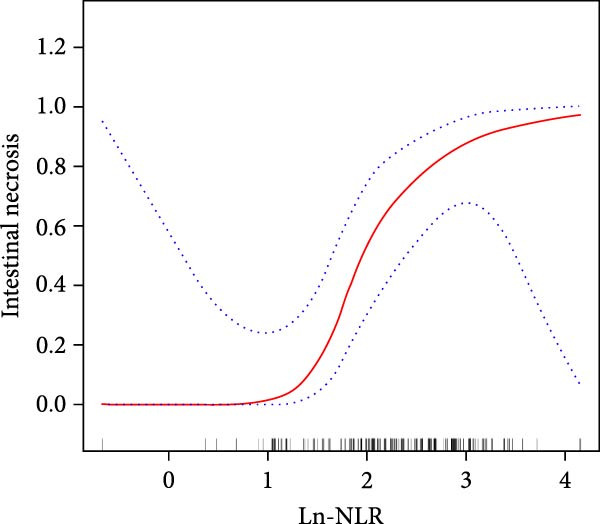
(A)

**Figure:**
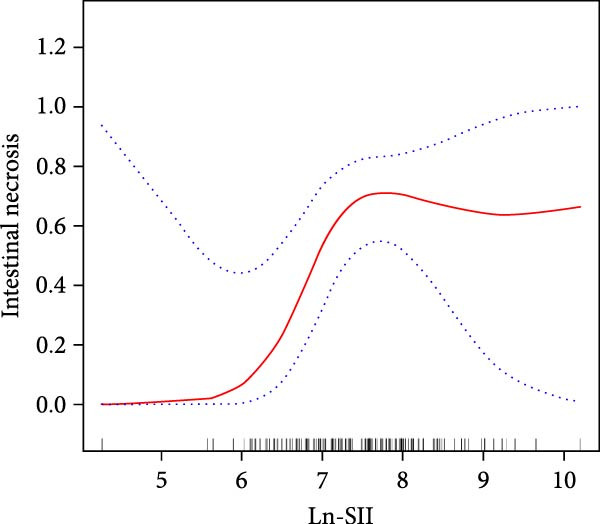
(B)

**Table 4:** Threshold effect analysis of the relationship between the Ln‐NLR, Ln‐SII, and intestinal necrosis in acute intestinal ischemia patients.

Threshold effect analysis	Intestinal necrosis OR (95% CI) *p*‐value
Ln‐NLR	
Inflection point of Ln‐NLR (K)	1.78
<K slope	1.005 (0.358–2.872) 0.980
> K slope	5.634 (1.503–21.126) 0.010
Log‐likelihood ratio test	*p* = 0.006
Ln‐SII	
Inflection point of Ln‐SII (K)	7.32
<K slope	38.565 (3.032–490.497) 0.005
>K slope	0.507 (0.104–2.471) 0.401
Log‐likelihood ratio test	*p* = 0.005

*Note:* Age, gender, BMI, hypertension, diabetes, coronary heart disease, etiology, onset time, WBC, NLR, SII, sodium, glucose, albumin, Scr, LDH, CRP, and D‐dimer were adjusted. *p* < 0.05 is indicated in bold.

### 3.5. Sensitivity Analysis

First, we performed multiple subgroup analyses and interaction tests based on various covariates were adopted for evaluating the robustness of the relationship between NLR, SII, and intestinal necrosis, and for identifying potential population differences. The results showed that in all subgroups, patients with NLR ≥ 8.85 or SII ≥ 1492.11 had a higher risk of intestinal necrosis (OR > 1), and the difference was significant in most subgroups, indicating that the conclusions of this study are robust (Table 5). Notably, a significant interaction was observed between NLR, intestinal necrosis, and coronary heart disease (*p* interaction = 0.016). In participants without coronary heart disease, we observed a positive correlation between NLR levels and intestinal necrosis (OR = 14.94, 95% CI = 6.01–37.09, *p*  < 0.001), but this association became insignificant in patients with coronary heart disease (Table 5). This suggests that NLR ≥ 8.85 is more likely to increase the risk of intestinal necrosis in patients without coronary heart disease.

**Table 5:** Subgroup analysis of the correlation between NLR, SII and intestinal necrosis in acute intestinal ischemia patients.

Subgroup	OR (95% CI) *p*‐value					
	NLR <8.85	NLR ≥8.85	*p* interaction	SII <1492.11	SII ≥1492.11	*p* interaction
Age	—	—	0.284	—	—	0.396
<60	Ref	20.00 (3.82–104.60) **<0.001**	—	Ref	11.56 (2.41–55.39) **0.002**	—
≥60	Ref	7.17 (2.94–17.47) **<0.001**	—	Ref	5.32 (2.24–12.65) **<0.001**	—
Gender	—	—	0.940	—	—	0.299
Male	Ref	9.67 (3.69–25.29) **<0.001**	—	Ref	4.90 (2.01–11.96) **<0.001**	—
Female	Ref	9.07 (2.31–35.65) **0.002**	—	Ref	12.00 (2.86–50.31) **<0.001**	—
Hypertension	—	—	0.107	—	—	0.817
No	Ref	14.28 (5.40–37.78) **<0.001**	—	Ref	6.16 (2.57–14.78) **<0.001**	—
Yes	Ref	3.40 (0.80–14.44) 0.097	—	Ref	7.60 (1.61–35.91) **0.010**	—
Diabetes	—	—	0.450	—	—	0.927
No	Ref	8.50 (3.72–19.42) **<0.001**	—	Ref	6.67 (2.95–15.04) **<0.001**	—
Yes	Ref	24.50 (1.79–336.22) **0.017**	—	Ref	6.00 (0.72–49.84) 0.097	—
Coronary heart disease	—	—	**0.016**	—	—	0.933
No	Ref	14.94 (6.01–37.09) **<0.001**	—	Ref	6.41 (2.81–14.61) **<0.001**	—
Yes	Ref	1.40 (0.26–7.58) 0.696	—	Ref	7.00 (1.07–45.90) **0.043**	—
Etiology	—	—	0.650	—	—	0.903
Nonvascular	Ref	12.54 (4.92–31.95) **<0.001**	—	Ref	8.06 (3.31–19.65) **<0.001**	—
Vascular	Ref	6.86 (0.60–77.98) 0.121	—	Ref	6.86 (0.60–77.98) 0.121	—
Onset time (h)	—	—	0.375	—	—	0.752
≤12	Ref	5.72 (1.60–20.45) **0.007**	—	Ref	8.33 (1.99–34.87) **0.004**	—
>12	Ref	12.00 (4.29–33.54) **<0.001**	—	Ref	6.31 (2.42–16.45) **<0.001**	—
BMI (kg/m^2^)	—	—	0.920	—	—	0.231
<25	Ref	10.31 (4.06–26.19) **<0.001**	—	Ref	5.07 (2.15–11.97) **<0.001**	—
≥25	Ref	9.39 (1.92–45.80) **0.006**	—	Ref	16.00 (3.00–85.30) **0.001**	—
D‐dimer (mg/L)	—	—	0.441	—	—	0.629
≤0.5	Ref	3.67 (0.27–49.29) 0.327	—	Ref	3.86 (0.33–45.56) 0.284	—
>0.5	Ref	10.77 (4.53–25.58) **<0.001**	—	Ref	7.33 (3.20–16.79) **<0.001**	—

*Note:* *p* < 0.05 is indicated in bold.

Subsequently, we performed PSM analysis to verify the relationship between NLR, SII, and intestinal necrosis. After PSM analysis, there were no differences in clinical data (Supporting Information: Tables S4 and S5). Then, we analyzed the association between NLR, SII, and intestinal necrosis after PSM analysis. Logistic regression analysis showed that compared with the NLR < 8.85 group, the risk of intestinal necrosis in the NLR ≥ 8.85 group increased (OR = 4.444, 95% CI = 1.076–18.355, *p* = 0.039). Compared with the SII < 1492.11 group, the risk of intestinal necrosis in the SII ≥ 1492.11 group increased (OR = 6.679, 95% CI = 1.576–28.293, *p* = 0.010) (Supporting Information: Table S6). These sensitivity analyses indicate that the positive association between NLR, SII, and intestinal necrosis in acute intestinal ischemia patients is stable.

## 4. Discussion

In this study, we analyzed the clinical data of 138 patients with different types of acute intestinal ischemia and identified vascular intestinal ischemia, NLR ≥ 8.85, SII ≥ 1492.11, CRP, and D‐dimer > 0.5 mg/L as independent risk factors for intestinal necrosis. However, PLR was not associated with the occurrence of intestinal necrosis. After adjusting for all covariates, the levels of Ln‐NLR and Ln‐SII in patients were still significantly positively correlated with intestinal necrosis, showing a nonlinear dose‐response relationship. Threshold effect analysis showed that when NLR > 5.93 or SII < 1510.20, the risk of intestinal necrosis significantly increased. To our knowledge, this study provides one of the first comprehensive evaluations of NLR, PLR, and SII in patients encompassing all major types of acute intestinal ischemia, and establishes their quantitative thresholds for predicting intestinal necrosis. While previous studies have examined NLR or PLR in selected subtypes of intestinal ischemia, our study extends these findings by introducing SII and demonstrating its reliable association and a robust, independent dose–response relationship across all major forms of acute intestinal ischemia, even after full covariate adjustment. These results highlight the clinical utility of SII and NLR as accessible and integrative inflammatory biomarkers in assessing intestinal viability.

Acute intestinal ischemia carries a dire prognosis, particularly once necrosis sets in, underscoring the critical need for early risk stratification [5, 33]. While the association between inflammation and intestinal necrosis is conceptually established, clinically actionable, and quantifiable biomarkers that capture this risk across all etiologies are lacking [34, 35]. Our study directly addresses this gap by identifying a panel of independent risk factors—vascular etiology, NLR ≥ 8.85, SII ≥ 1492.11, CRP, and D‐dimer > 0.5 mg/L—that collectively refine the prediction of intestinal necrosis in acute intestinal ischemia patient cohort and provide a quantitative foundation for a new diagnostic approach. Vascular intestinal ischemia includes mesenteric artery embolism, arterial or venous thrombosis, and nonocclusive mesenteric ischemia. These patients often lack typical clinical manifestations in the early stages, and due to the current limitations of early diagnostic techniques, they are often misdiagnosed, with most patients being diagnosed only after intestinal necrosis has already occurred [36]. These may be important reasons for the high risk of intestinal necrosis in vascular intestinal ischemia patients. Recent research has shown that CRP > 15 mg/L is an independent risk factor for extensive (≥100 cm) irreversible intestinal necrosis in acute intestinal ischemia patients [37]. Chen et al. [38] found that acute intestinal ischemia patients with intestinal necrosis often have elevated D‐dimer levels. These findings are consistent with our results.

NLR is a simple and widely used inflammatory marker, and its increase is often associated with systemic inflammatory responses, immune imbalance, and the prognosis of various diseases [39–41]. In this study, we demonstrated that NLR ≥ 8.85 is an independent risk factor for intestinal necrosis in acute intestinal ischemia patients. After adjusting for all covariates, the Ln‐NLR level in patients was still significantly positively correlated with intestinal necrosis. While previous studies, such as that by Wang et al. and Beji et al., established a correlation between NLR and intestinal necrosis in specific subtypes like arterial embolism and thrombosis and incarcerated hernia, our study significantly expands this understanding in several key aspects [21, 22]. First, we validate the prognostic value of NLR across all major types of acute intestinal ischemia, including intestinal adhesions, torsion, strangulated obstruction, incarcerated hernias, mesenteric artery embolism, arterial or venous thrombosis, and nonocclusive causes, demonstrating its utility as a universal inflammatory marker irrespective of the initial ischemic etiology. Second, and more importantly, we move beyond a simple correlation by establishing a precise, high‐threshold (≥8.85), which more accurately captures the intense systemic inflammatory burden required to tip the balance towards necrosis. Third, our analysis adjusted for multiple clinical covariates to minimize confounding and better define the independent effects of NLR on intestinal necrosis. The attenuated association of NLR in patients with coronary heart disease is a novel insight. This may be because patients with coronary artery disease typically exhibit chronic low‐grade systemic inflammation (e.g., atherosclerosis‐related inflammation), which could lead to elevated baseline NLR levels, thereby diminishing its predictive value for acute intestinal ischemia [42]. Second, coronary artery disease patients often present with systemic microcirculatory dysfunction (e.g., endothelial impairment) and arteriosclerosis in peripheral, mesenteric, and renal arteries. This may compromise intestinal tolerance, making them more susceptible to bowel necrosis, which in turn weakens the association between NLR and intestinal necrosis [43]. The results of subgroup analysis suggested that chronic baseline inflammation may mask the predictive signal of an acute inflammatory surge, thus refining the contexts in which NLR is most useful.

SII is a new comprehensive marker of inflammation and immune response, and its predictive ability for some diseases exceeds that of traditional indicators such as NLR and PLR [44, 45]. We found that SII ≥ 1492.11 is an independent risk factor for intestinal necrosis in acute intestinal ischemia patients. After adjusting for all covariates, the Ln‐SII level in patients was still significantly positively correlated with intestinal necrosis. In contrast to NLR, the association between SII and intestinal necrosis represents a novel finding with distinct pathophysiological implications. To our knowledge, this is the first study to identify SII as an independent predictor of intestinal necrosis in a heterogeneous cohort of acute intestinal ischemia patients. SII integrates neutrophils, platelets, and lymphocytes, thereby offering a more holistic reflection of the “immunothrombotic” state—a vicious cycle where inflammation promotes thrombosis in the microvasculature, and thrombosis further exacerbates inflammation and ischemia [46]. The stability of SII’s predictive power across all subgroups, unlike NLR, suggests it may be a more robust biomarker, less confounded by comorbidities like coronary heart disease. This may be because SII captures the platelet‐driven microvascular thrombosis that NLR misses.

The observed associations between elevated NLR/SII and intestinal necrosis can be interpreted in light of the inflammatory cascade triggered by ischemic injury. Intestinal ischemia induces epithelial barrier disruption and translocation of endotoxins, amplifying systemic neutrophil activation and cytokine release. This amplifies microvascular obstruction and reperfusion injury, forming a vicious cycle that promotes necrosis [47, 48]. The strong correlation between neutrophil‐dominant indices (NLR, SII) and necrosis risk in our study supports this pathophysiological mechanism, suggesting that neutrophil‐driven inflammation rather than platelet–lymphocyte imbalance plays a dominant role in the transition from ischemia to irreversible necrosis. This also provides a plausible explanation for the lack of association we observed between PLR and intestinal necrosis. PLR fails to account for the paramount role of neutrophils as the primary mediators of acute ischemic injury.

Beyond confirming inflammatory activation, our findings provide quantitative evidence for risk stratification, which have not been previously reported. We identified that when NLR > 5.93 or SII < 1510.20, the risk of intestinal necrosis significantly increased. Interestingly, the threshold analysis revealed that the risk of intestinal necrosis increased when SII was below ~1510, but appeared to decline at extremely high SII levels. This nonlinear pattern may reflect several phenomena. First, at very high systemic inflammation levels, pathophysiologic processes may reach a plateau—patients are already in advanced systemic inflammatory, immune exhaustion or immunosuppression states, in which further elevation of SII no longer proportionally increases necrosis risk [49, 50]. Second, patients with extremely high SII values are more likely to develop multiorgan failure or receive early surgical or intensive interventions, potentially reducing the observed incidence of necrosis. These findings provide clinicians with practical reference points for risk stratification and early decision‐making. For patients suspected of necrosis, these thresholds may help identify those who require urgent surgical, antibiotic, or revascularization therapy. While our results support the clinical relevance of NLR and SII, larger prospective multicenter studies are required to confirm their predictive thresholds and to determine their utility in clinical practice. Importantly, NLR and SII should not replace but rather complement existing diagnostic modalities. Integrating these parameters with imaging and biochemical indicators may refine existing diagnostic algorithms for acute intestinal ischemia. We propose a potential integrated algorithm: elevated NLR or SII may prompt expedited CTA in equivocal cases, and normal values could help rule out necrosis in low‐risk patients. This synergistic approach may reduce unnecessary radiation exposure while maintaining diagnostic accuracy. This approach transforms previously descriptive biomarkers into actionable, threshold‐based tools that can guide real‐time clinical decisions.

It is undeniable that this study has some limitations: First, due to the low incidence of acute intestinal ischemia, the number of cases included in this study may be relatively small, which may amplify OR estimates in logistic regression; Second, this study is single‐center and retrospective, which may lead to bias and limit the generalizability of the results. Future multicenter, large‐scale prospective studies are needed to validate our conclusions. Third, although we adjusted for some confounding factors related to intestinal necrosis, we cannot completely rule out the influence of other potential confounding factors on the association between NLR, SII, and intestinal necrosis.

## 5. Conclusion

Collectively, this study delineates a distinct risk profile for intestinal necrosis, centered on quantifiable systemic inflammation. We move beyond established correlations by defining precise, clinically relevant thresholds for NLR and SII and introducing the concept of a nonlinear relationship with necrosis risk. These findings provide a compelling framework for integrating these readily available biomarkers into clinical decision‐making to guide earlier intervention and improve patient outcomes.

## Ethics Statement

The study was conducted in accordance with the Declaration of Helsinki, and approved by the Ethics Committee of the First Hospital of Qinhuangdao (No. 2025 K‐157‐01).

## Consent

Patient consent was waived due to the retrospective nature of the study. All data used in this manuscript were anonymized.

## Disclosure

All authors have read and agreed to the published version of the manuscript.

## Conflicts of Interest

The authors declare no conflicts of interest.

## Author Contributions

Conceptualization: Yu Tian and Feifan Wang. Methodology: Yu Tian, Feifan Wang, Mingshuo Zhang, Rui Ding, and Yimin Wang. Validation, formal analysis, investigation: Yu Tian, Feifan Wang, Mingshuo Zhang, and Rui Ding. Writing – original draft and visualization: Yu Tian and Feifan Wang. Writing – review and editing: Yimin Wang. Supervision, project administration, funding acquisition: Yimin Wang. Yu Tian and Feifan Wang contributed equally to this work.

## Funding

This study was funded by the Qinhuangdao Science and Technology Research and Development Program, Grant 202201B030.

## Supporting Information

Additional supporting information can be found online in the Supporting Information section.

## Supporting information


**Supporting Information** Figure S1: The distribution of NLR (A) and SII (B). The distribution of ln‐transformed NLR (C) and ln‐transformed SII (D). Table S1: Characteristics of the patients according to the quartiles of Ln‐NLR. Table S2: Characteristics of the patients according to the quartiles of Ln‐SII. Table S3: The VIF values of all variables included in the multivariate logistic regression model. Table S4: Clinical characteristics of intestinal necrosis patients with NLR < 8.85 and NLR ≥ 8.85 before and after PSM. Table S5: Clinical characteristics of intestinal necrosis patients with SII < 1492.11 and SII ≥ 1492.11 before and after PSM. Table S6: Logistic regression analysis for the association between NLR, SII, and intestinal necrosis in acute intestinal ischemia patients after PSM.

## Data Availability

The original contributions presented in the study are included in the article, further inquiries can be directed to the corresponding author.
