# Supplementary material for: Comprehensive Analysis of the Correlation Between Immune‐Inflammatory Biomarkers and Intestinal Necrosis in Patients With Acute Intestinal Ischemia
Source: Mediators Inflamm. 2025 Dec 29;2025:8701105. doi: 10.1155/mi/8701105 (PMC12767384; doi:10.1155/mi/8701105)
Supplement: Supplementary file 1 — Supporting Information Figure S1: The distribution of NLR (A) and SII (B). The distribution of ln‐transformed NLR (C) and ln‐transformed SII (D). Table S1: Characteristics of the patients according to the quartiles of Ln‐NLR. Table S2: Characteristics of the patients according to the quartiles of Ln‐SII. Table S3: The VIF values of all variables included in the multivariate logistic regression model. Table S4: Clinical characteristics of intestinal necrosis patients with NLR < 8.85 and NLR ≥ 8.85 before and after PSM. Table S5: Clinical characteristics of intestinal necrosis patients with SII < 1492.11 and SII ≥ 1492.11 before and after PSM. Table S6: Logistic regression analysis for the association between NLR, SII, and intestinal necrosis in acute intestinal ischemia patients after PSM. [file MI-2025-8701105-s001.docx]

**Comprehensive analysis of the correlation between** **immune-inflammatory biomarkers and intestinal necrosis in patients with acute intestinal ischemia**

**Yu Tian^1, †^, Feifan Wang^1, †^, Mingshuo Zhang^1,2^, Rui Ding^1,3^, Yimin Wang^4,*^**

1 Department of Hand & Foot Surgery, First Hospital of Qinhuangdao, Qinhuangdao 066000, China.

2 Hebei North University, Zhangjiakou 075000, China.

3 Hebei Medical University, Shijiazhuang 050017, China.

4 Department of General Surgery, First Hospital of Qinhuangdao, Qinhuangdao 066000, China.

**^*^** Correspondence: Yimin Wang, drwangyimin@126.com

**^†^** These authors contributed equally to this work.


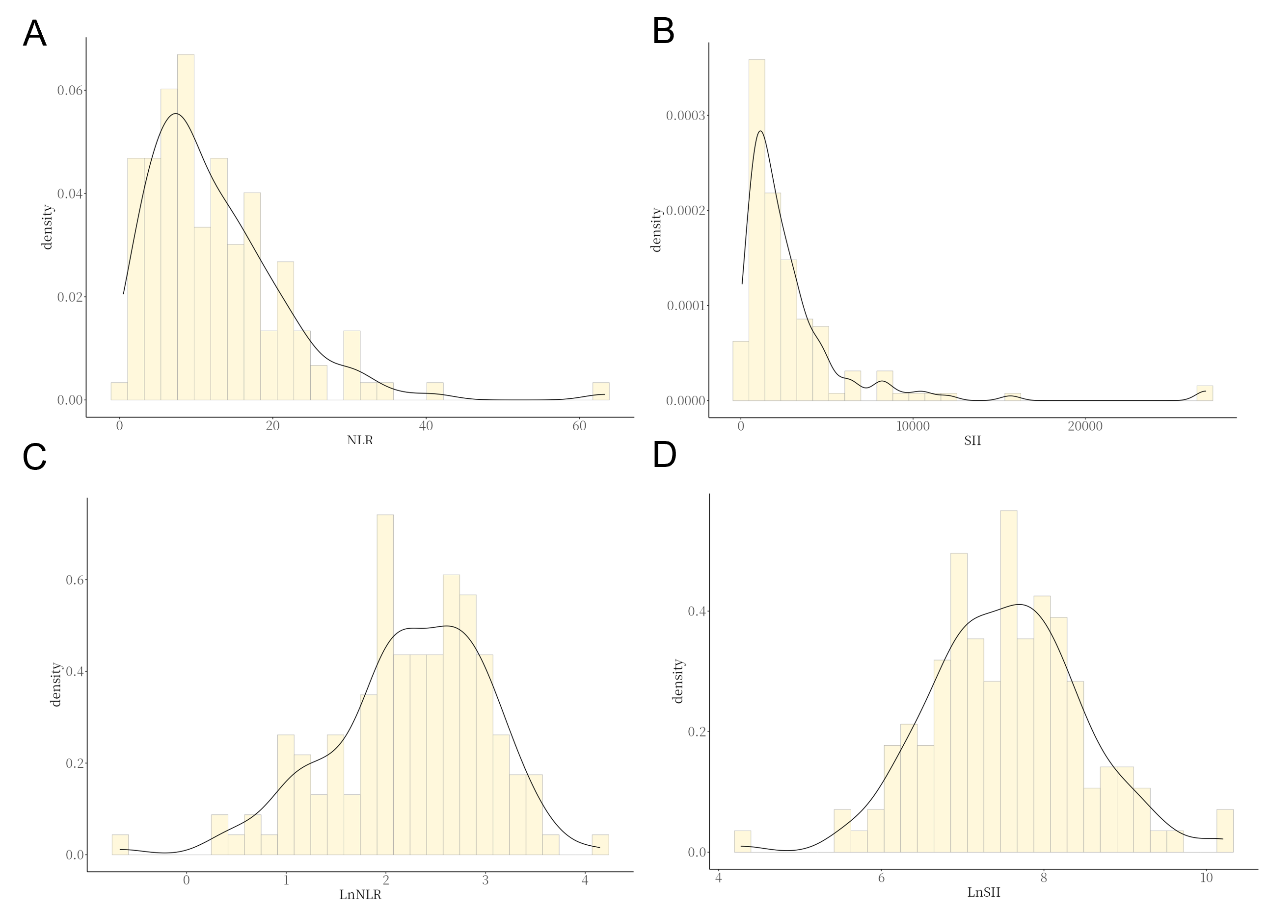


Figure S1 The distribution of NLR (A) and SII (B). The distribution of ln-transformed NLR (C) and ln-transformed SII (D).

Table S1 Characteristics of the patients according to the quartiles of Ln-NLR.

| Characteristic | Ln-NLR quartiles | | | | *P* |
| --- | --- | --- | --- | --- | --- |
|  | Q1 (n = 35) | Q2 (n = 34) | Q3 (n = 34) | Q4 (n = 35) |  |
| Age | 70.00 (58.00,75.00) | 68.50 (56.00,76.75) | 61.50 (55.25,73.75) | 70.00 (63.00,79.00) | 0.480 |
| Gender |  |  |  |  | 0.478 |
| Male | 22 (62.86%) | 21 (61.76%) | 24 (70.59%) | 27 (77.14%) |  |
| Female | 13 (37.14%) | 13 (38.24%) | 10 (29.41%) | 8 (22.86%) |  |
| Hypertension |  |  |  |  | 0.285 |
| No | 29 (82.86%) | 25 (73.53%) | 26 (76.47%) | 22 (62.86%) |  |
| Yes | 6 (17.14%) | 9 (26.47%) | 8 (23.53%) | 13 (37.14%) |  |
| Diabetes |  |  |  |  | 0.689 |
| No | 29 (82.86%) | 31 (91.18%) | 31 (91.18%) | 30 (85.71%) |  |
| Yes | 6 (17.14%) | 3 (8.82%) | 3 (8.82%) | 5 (14.29%) |  |
| Coronary heart disease |  |  |  |  | 0.869 |
| No | 31 (88.57%) | 28 (82.35%) | 28 (82.35%) | 29 (82.86%) |  |
| Yes | 4 (11.43%) | 6 (17.65%) | 6 (17.65%) | 6 (17.14%) |  |
| Etiology |  |  |  |  | 0.110 |
| Non-vascular | 32 (91.43%) | 25 (73.53%) | 29 (85.29%) | 25 (71.43%) |  |
| Vascular | 3 (8.57%) | 9 (26.47%) | 5 (14.71%) | 10 (28.57%) |  |
| Onset time (h) |  |  |  |  | 0.099 |
| ≤ 12 | 16 (45.71%) | 12 (35.29%) | 15 (44.12%) | 7 (20.00%) |  |
| > 12 | 19 (54.29%) | 22 (64.71%) | 19 (55.88%) | 28 (80.00%) |  |
| BMI (kg/m^2^) | 22.50 (22.10,26.70) | 22.10 (20.25,24.73) | 22.10 (20.70,23.92) | 22.10 (20.00,22.10) | 0.142 |
| Temperature (℃) | 36.50 (36.20,36.60) | 36.50 (36.30,36.68) | 36.45 (36.30,36.68) | 36.50 (36.30,36.95) | 0.346 |
| WBC (10^9^/L) | 5.99 (3.58,8.29) | 7.79 (6.52,11.54) | 10.33 (7.49,13.13) | 12.16 (10.27,15.58) | **<.001** |
| PLR | 158.00 (128.17,275.33) | 208.72 (139.05,274.56) | 249.19 (163.85,358.05) | 377.94 (246.65,532.38) | **<.001** |
| SII (*10^9^) | 699.77 (478.50,918.18) | 1546.36 (1167.56,1965.60) | 2714.65 (2055.96,3345.96) | 4713.80 (3205.40,8238.20) | **<.001** |
| HGB (g/L) | 124.49 ± 27.51 | 130.21 ± 22.57 | 125.00 ± 28.53 | 128.11 ± 32.14 | 0.811 |
| PLT (10^9^/L) | 187.00 (156.00,245.50) | 202.50 (154.75,248.00) | 200.50 (154.00,237.75) | 249.00 (149.50,317.50) | 0.393 |
| Potassium (mmol/L) |  |  |  |  | 0.399 |
| 3.5-5.5 | 28 (80.00%) | 25 (73.53%) | 25 (73.53%) | 28 (80.00%) |  |
| < 3.5 | 5 (14.29%) | 8 (23.53%) | 9 (26.47%) | 4 (11.43%) |  |
| > 5.5 | 2 (5.71%) | 1 (2.94%) | 0 (0.00%) | 3 (8.57%) |  |
| Sodium (mmol/L) |  |  |  |  | 0.192 |
| ≥ 135 | 33 (94.29%) | 30 (88.24%) | 28 (82.35%) | 27 (77.14%) |  |
| < 135 | 2 (5.71%) | 4 (11.76%) | 6 (17.65%) | 8 (22.86%) |  |
| Glucose (mmol/L) | 6.54 (5.70,9.36) | 7.04 (6.64,9.06) | 8.12 (7.07,10.05) | 9.08 (6.40,11.89) | 0.147 |
| Albumin (g/L) | 35.90 (29.55,41.76) | 34.60 (27.35,42.17) | 34.95 (29.02,42.40) | 30.90 (23.45,37.70) | 0.131 |
| Scr (μmol/L) | 67.60 (56.45,82.40) | 68.00 (53.75,75.15) | 85.20 (57.60,150.72) | 99.20 (64.95,168.45) | **0.009** |
| LDH (U/L) | 204.00 (164.50,241.00) | 196.50 (177.00,235.25) | 258.00 (197.25,328.00) | 304.00 (206.00,426.00) | **<.001** |
| CRP (mg/L) | 21.26 (7.70,45.98) | 40.70 (2.38,122.04) | 94.83 (13.28,206.91) | 99.04 (25.39,176.04) | **0.011** |
| D-dimer (mg/L) |  |  |  |  | 0.416 |
| ≤ 0.5 | 8 (22.86%) | 4 (11.76%) | 5 (14.71%) | 3 (8.57%) |  |
| > 0.5 | 27 (77.14%) | 30 (88.24%) | 29 (85.29%) | 32 (91.43%) |  |
| Intestinal necrosis |  |  |  |  | **<.001** |
| No | 31 (88.57%) | 18 (52.94%) | 10 (29.41%) | 6 (17.14%) |  |
| Yes | 4 (11.43%) | 16 (47.06%) | 24 (70.59%) | 29 (82.86%) |  |

Table S2 Characteristics of the patients according to the quartiles of Ln-SII.

| Characteristic | Ln-SII quartiles | | | | *P* |
| --- | --- | --- | --- | --- | --- |
|  | Q1 (n = 35) | Q2 (n = 34) | Q3 (n = 34) | Q4 (n = 35) |  |
| Age | 70.00 (61.50,75.50) | 68.00 (55.25,73.75) | 62.00 (58.25,72.50) | 73.00 (56.00,80.50) | 0.234 |
| Gender |  |  |  |  | 0.637 |
| Male | 21 (60.00%) | 23 (67.65%) | 25 (73.53%) | 25 (71.43%) |  |
| Female | 14 (40.00%) | 11 (32.35%) | 9 (26.47%) | 10 (28.57%) |  |
| Hypertension |  |  |  |  | 0.309 |
| No | 26 (74.29%) | 28 (82.35%) | 26 (76.47%) | 22 (62.86%) |  |
| Yes | 9 (25.71%) | 6 (17.65%) | 8 (23.53%) | 13 (37.14%) |  |
| Diabetes |  |  |  |  | 0.171 |
| No | 28 (80.00%) | 30 (88.24%) | 33 (97.06%) | 30 (85.71%) |  |
| Yes | 7 (20.00%) | 4 (11.76%) | 1 (2.94%) | 5 (14.29%) |  |
| Coronary heart disease |  |  |  |  | 0.456 |
| No | 29 (82.86%) | 29 (85.29%) | 31 (91.18%) | 27 (77.14%) |  |
| Yes | 6 (17.14%) | 5 (14.71%) | 3 (8.82%) | 8 (22.86%) |  |
| Etiology |  |  |  |  | 0.925 |
| Non-vascular | 29 (82.86%) | 27 (79.41%) | 28 (82.35%) | 27 (77.14%) |  |
| Vascular | 6 (17.14%) | 7 (20.59%) | 6 (17.65%) | 8 (22.86%) |  |
| Onset time (h) |  |  |  |  | 0.340 |
| ≤ 12 | 17 (48.57%) | 10 (29.41%) | 12 (35.29%) | 11 (31.43%) |  |
| > 12 | 18 (51.43%) | 24 (70.59%) | 22 (64.71%) | 24 (68.57%) |  |
| BMI (kg/m^2^) | 22.10 (20.00,26.55) | 22.30 (21.57,25.25) | 21.05 (19.62,22.73) | 22.10 (21.05,25.00) | 0.181 |
| Temperature (℃) | 36.50 (36.25,36.65) | 36.50 (36.30,36.68) | 36.50 (36.30,36.68) | 36.50 (36.30,36.80) | 0.838 |
| WBC (10^9^/L) | 5.99 (3.68,8.17) | 8.87 (6.21,11.69) | 8.12 (6.38,10.38) | 12.46 (11.00,16.27) | **<.001** |
| NLR | 3.94 (2.85,6.03) | 7.88 (6.94,10.40) | 13.67 (9.89,14.68) | 20.57 (16.40,25.96) | **<.001** |
| PLR | 150.69 (119.23,236.37) | 189.92 (128.77,242.38) | 260.51 (200.03,397.19) | 367.24 (313.64,532.38) | **<.001** |
| HGB (g/L) | 124.74 ± 27.75 | 125.03 ± 22.59 | 125.74 ± 33.60 | 132.17 ± 26.41 | 0.643 |
| PLT (10^9^/L) | 167.00 (113.50,205.00) | 176.00 (142.00,224.50) | 209.00 (151.50,248.00) | 279.00 (237.00,350.00) | **<.001** |
| Potassium (mmol/L) |  |  |  |  | 0.305 |
| 3.5-5.5 | 28 (80.00%) | 23 (67.65%) | 27 (79.41%) | 28 (80.00%) |  |
| < 3.5 | 4 (11.43%) | 10 (29.41%) | 7 (20.59%) | 5 (14.29%) |  |
| > 5.5 | 3 (8.57%) | 1 (2.94%) | 0 (0.00%) | 2 (5.71%) |  |
| Sodium (mmol/L) |  |  |  |  | 0.071 |
| ≥ 135 | 33 (94.29%) | 31 (91.18%) | 25 (73.53%) | 29 (82.86%) |  |
| < 135 | 2 (5.71%) | 3 (8.82%) | 9 (26.47%) | 6 (17.14%) |  |
| Glucose (mmol/L) | 6.54 (5.82,8.95) | 7.21 (6.72,8.13) | 7.89 (6.62,9.65) | 10.39 (6.93,12.02) | **0.006** |
| Albumin (g/L) | 35.10 (26.10,40.60) | 32.85 (26.82,41.15) | 35.40 (27.55,43.85) | 35.20 (30.15,41.30) | 0.862 |
| Scr (μmol/L) | 69.10 (63.80,94.05) | 67.10 (51.67,76.83) | 77.75 (57.60,142.93) | 79.30 (56.65,161.00) | 0.196 |
| LDH (U/L) | 215.00 (169.50,269.00) | 197.50 (176.00,237.75) | 209.00 (191.75,271.00) | 286.00 (203.00,383.00) | **0.014** |
| CRP (mg/L) | 32.75 (7.70,76.99) | 33.61 (2.38,95.93) | 97.97 (26.62,191.80) | 81.48 (16.50,176.04) | 0.066 |
| D-dimer (mg/L) |  |  |  |  | 0.744 |
| ≤ 0.5 | 5 (14.29%) | 6 (17.65%) | 3 (8.82%) | 6 (17.14%) |  |
| > 0.5 | 30 (85.71%) | 28 (82.35%) | 31 (91.18%) | 29 (82.86%) |  |
| Intestinal necrosis |  |  |  |  | **<.001** |
| No | 28 (80.00%) | 19 (55.88%) | 10 (29.41%) | 8 (22.86%) |  |
| Yes | 7 (20.00%) | 15 (44.12%) | 24 (70.59%) | 27 (77.14%) |  |

Table S3. The VIF values of all variables included in the multivariate logistic regression model.

| Variable | VIF value | Variable | VIF value |
| --- | --- | --- | --- |
| Age | 1.36 | Ln-NLR | 3.42 |
| Gender | 1.29 | Ln-SII | 3.91 |
| BMI | 1.44 | Sodium | 1.37 |
| Hypertension | 1.53 | Glucose | 1.41 |
| Diabetes | 1.98 | Albumin | 1.56 |
| Coronary heart disease | 1.92 | Scr | 1.63 |
| Etiology | 1.46 | LDH | 1.31 |
| Onset time | 1.44 | CRP | 1.59 |
| WBC | 2.71 | D-dimer | 1.26 |

Table S4 Clinical characteristics of intestinal necrosis patients with NLR < 8.85 and NLR ≥ 8.85 before and after PSM.

| Characteristic | Before PSM | | | After PSM | | |
| --- | --- | --- | --- | --- | --- | --- |
|  | NLR < 8.85  (n = 61) | NLR ≥ 8.85  (n = 77) | *P* | NLR < 8.85  (n = 25) | NLR ≥ 8.85  (n = 14) | *P* |
| Age | 68.00 (56.00, 75.00) | 69.00 (60.00, 78.00) | 0.378 | 72.00 (64.00, 77.00) | 66.00 (54.75, 73.75) | 0.500 |
| Gender |  |  | 0.192 |  |  | 1.000 |
| Male | 38 (62.30%) | 56 (72.73%) |  | 17 (68.00%) | 10 (71.43%) |  |
| Female | 23 (37.70%) | 21 (27.27%) |  | 8 (32.00%) | 4 (28.57%) |  |
| Hypertension |  |  | 0.455 |  |  | 1.000 |
| No | 47 (77.05%) | 55 (71.43%) |  | 18 (72.00%) | 10 (71.43%) |  |
| Yes | 14 (22.95%) | 22 (28.57%) |  | 7 (28.00%) | 4 (28.57%) |  |
| Diabetes |  |  | 0.438 |  |  | 1.000 |
| No | 52 (85.25%) | 69 (89.61%) |  | 22 (88.00%) | 12 (85.71%) |  |
| Yes | 9 (14.75%) | 8 (10.39%) |  | 3 (12.00%) | 2 (14.29%) |  |
| Coronary heart disease |  |  | 0.897 |  |  | 1.000 |
| No | 51 (83.61%) | 65 (84.42%) |  | 19 (76.00%) | 11 (78.57%) |  |
| Yes | 10 (16.39%) | 12 (15.58%) |  | 6 (24.00%) | 3 (21.43%) |  |
| Etiology |  |  | 0.403 |  |  | 0.723 |
| Non-vascular | 51 (83.61%) | 60 (77.92%) |  | 18 (72.00%) | 9 (64.29%) |  |
| Vascular | 10 (16.39%) | 17 (22.08%) |  | 7 (28.00%) | 5 (35.71%) |  |
| Onset time (h) |  |  | 0.081 |  |  | 1.000 |
| ≤ 12 | 27 (44.26%) | 23 (29.87%) |  | 10 (40.00%) | 5 (35.71%) |  |
| > 12 | 34 (55.74%) | 54 (70.13%) |  | 15 (60.00%) | 9 (64.29%) |  |
| BMI (kg/m^2^) | 22.50 (20.50, 25.70) | 22.10 (19.70, 22.70) | **0.013** | 22.10 (21.50, 23.40) | 22.10 (21.73, 24.27) | 0.988 |
| Temperature (℃) | 36.50 (36.20, 36.60) | 36.50 (36.30, 36.80) | 0.089 | 36.40 (36.20, 36.60) | 36.45 (36.20, 36.70) | 0.658 |
| WBC (10^9^/L) | 6.73 (5.14, 9.86) | 10.83 (8.57, 14.25) | **<.001** | 6.95 (5.36, 11.54) | 10.36 (7.26, 11.62) | 0.111 |
| PLR | 174.60 (130.65, 263.64) | 300.00 (182.88, 421.28) | **<.001** | 181.82 (150.00, 270.51) | 208.29 (140.03, 397.19) | 0.608 |
| SII (*10^9^) |  |  | **<.001** |  |  | 0.518 |
| < 1492.11 | 46 (75.41%) | 11 (14.29%) |  | 12 (48.00%) | 5 (35.71%) |  |
| ≥ 1492.11 | 15 (24.59%) | 66 (85.71%) |  | 13 (52.00%) | 9 (64.29%) |  |
| HGB (g/L) | 129.75 ± 25.56 | 124.71 ± 29.29 | 0.291 | 123.88 ± 24.36 | 122.29 ± 30.87 | 0.860 |
| PLT (10^9^/L) | 193.00 (158.00, 248.00) | 213.00 (150.00, 278.00) | 0.444 | 211.00 (135.00, 248.00) | 191.50 (86.00, 365.75) | 0.953 |
| Potassium (mmol/L) |  |  | 0.798 |  |  | 0.673 |
| 3.5-5.5 | 48 (78.69%) | 58 (75.32%) |  | 18 (72.00%) | 8 (57.14%) |  |
| < 3.5 | 10 (16.39%) | 16 (20.78%) |  | 5 (20.00%) | 4 (28.57%) |  |
| > 5.5 | 3 (4.92%) | 3 (3.90%) |  | 2 (8.00%) | 2 (14.29%) |  |
| Sodium (mmol/L) |  |  | 0.167 |  |  | 0.686 |
| ≥ 135 | 55 (90.16%) | 63 (81.82%) |  | 21 (84.00%) | 11 (78.57%) |  |
| < 135 | 6 (9.84%) | 14 (18.18%) |  | 4 (16.00%) | 3 (21.43%) |  |
| Glucose (mmol/L) | 7.03 (5.97, 9.29) | 8.09 (6.57, 10.67) | 0.056 | 7.04 (6.62, 10.93) | 8.59 (6.73, 10.62) | 0.529 |
| Albumin (g/L) | 37.30 (30.10, 43.40) | 32.70 (26.40, 39.40) | **0.026** | 32.90 (27.40, 41.20) | 35.10 (23.12, 39.38) | 0.404 |
| Scr (μmol/L) | 68.60 (55.70, 77.90) | 88.40 (57.60, 161.00) | **0.009** | 71.90 (53.70, 76.90) | 66.10 (48.45, 133.97) | 1.000 |
| LDH (U/L) | 197.00 (166.00, 238.00) | 260.00 (201.00, 348.00) | **<.001** | 199.00 (193.00, 251.00) | 232.50 (208.00, 483.25) | 0.095 |
| CRP (mg/L) | 32.75 (3.47, 58.29) | 99.04 (20.12, 195.80) | **<.001** | 41.61 (12.80, 108.30) | 68.14 (3.06, 140.75) | 0.895 |
| D-dimer (mg/L) |  |  | 0.124 |  |  | 1.000 |
| ≤ 0.5 | 12 (19.67%) | 8 (10.39%) |  | 3 (12.00%) | 2 (14.29%) |  |
| > 0.5 | 49 (80.33%) | 69 (89.61%) |  | 22 (88.00%) | 12 (85.71%) |  |

Table S5 Clinical characteristics of intestinal necrosis patients with SII < 1492.11 and SII ≥ 1492.11 before and after PSM.

| Characteristic | Before PSM | | | After PSM | | |
| --- | --- | --- | --- | --- | --- | --- |
|  | SII < 1492.11  (n = 57) | SII≥ 1492.11  (n = 81) | *P* | SII < 1492.11  (n = 24) | SII ≥ 1492.11  (n = 15) | *P* |
| Age | 69.00 (58.00, 75.00) | 67.00 (58.00, 77.00) | 0.878 | 71.00 (67.75, 77.50) | 73.00 (57.00, 77.50) | 0.885 |
| Gender |  |  | 0.498 |  |  | 1.000 |
| Male | 37 (64.91%) | 57 (70.37%) |  | 16 (66.67%) | 10 (66.67%) |  |
| Female | 20 (35.09%) | 24 (29.63%) |  | 8 (33.33%) | 5 (33.33%) |  |
| Hypertension |  |  | 0.462 |  |  | 0.376 |
| No | 44 (77.19%) | 58 (71.60%) |  | 19 (79.17%) | 14 (93.33%) |  |
| Yes | 13 (22.81%) | 23 (28.40%) |  | 5 (20.83%) | 1 (6.67%) |  |
| Diabetes |  |  | 0.298 |  |  | 0.686 |
| No | 48 (84.21%) | 73 (90.12%) |  | 19 (79.17%) | 13 (86.67%) |  |
| Yes | 9 (15.79%) | 8 (9.88%) |  | 5 (20.83%) | 2 (13.33%) |  |
| Coronary heart disease |  |  | 0.666 |  |  | 1.000 |
| No | 47 (82.46%) | 69 (85.19%) |  | 20 (83.33%) | 13 (86.67%) |  |
| Yes | 10 (17.54%) | 12 (14.81%) |  | 4 (16.67%) | 2 (13.33%) |  |
| Etiology |  |  | 0.616 |  |  | 1.000 |
| Non-vascular | 47 (82.46%) | 64 (79.01%) |  | 21 (87.50%) | 13 (86.67%) |  |
| Vascular | 10 (17.54%) | 17 (20.99%) |  | 3 (12.50%) | 2 (13.33%) |  |
| Onset time (h) |  |  | 0.398 |  |  | 0.496 |
| ≤ 12 | 23 (40.35%) | 27 (33.33%) |  | 10 (41.67%) | 4 (26.67%) |  |
| > 12 | 34 (59.65%) | 54 (66.67%) |  | 14 (58.33%) | 11 (73.33%) |  |
| BMI (kg/m^2^) | 22.10 (20.08, 25.40) | 22.10 (20.50, 24.20) | 0.501 | 22.10 (19.05, 25.18) | 22.50 (20.45, 24.90) | 0.772 |
| Temperature (℃) | 36.50 (36.20, 36.70) | 36.50 (36.30, 36.70) | 0.618 | 36.50 (36.38, 36.60) | 36.50 (36.30, 36.60) | 0.704 |
| WBC (10^9^/L) | 6.76 (4.77, 9.86) | 10.72 (7.80, 13.46) | **<.001** | 6.67 (5.63, 9.39) | 7.39 (6.55, 10.95) | 0.260 |
| PLR | 156.67 (115.83, 246.48) | 300.00 (201.20, 419.64) | **<.001** | 147.14 (117.42, 241.18) | 216.22 (158.24, 291.35) | 0.097 |
| NLR |  |  | **<.001** |  |  | 1.000 |
| < 8.85 | 46 (80.70%) | 15 (18.52%) |  | 15 (62.50%) | 9 (60.00%) |  |
| ≥ 8.85 | 11 (19.30%) | 66 (81.48%) |  | 9 (37.50%) | 6 (40.00%) |  |
| HGB (g/L) | 122.93 ± 26.18 | 129.77 ± 28.58 | 0.155 | 124.17 ± 20.44 | 125.40 ± 33.39 | 0.887 |
| PLT (10^9^/L) | 167.00 (116.00, 194.00) | 237.00 (187.00, 283.00) | **<.001** | 185.00 (108.50, 258.50) | 226.00 (192.00, 256.50) | 0.214 |
| Potassium (mmol/L) |  |  | 0.895 |  |  | 1.000 |
| 3.5-5.5 | 43 (75.44%) | 63 (77.78%) |  | 17 (70.83%) | 12 (80.00%) |  |
| < 3.5 | 11 (19.30%) | 15 (18.52%) |  | 6 (25.00%) | 3 (20.00%) |  |
| > 5.5 | 3 (5.26%) | 3 (3.70%) |  | 1 (4.17%) | 0 (0.00%) |  |
| Sodium (mmol/L) |  |  | **0.036** |  |  | 1.000 |
| ≥ 135 | 53 (92.98%) | 65 (80.25%) |  | 22 (91.67%) | 14 (93.33%) |  |
| < 135 | 4 (7.02%) | 16 (19.75%) |  | 2 (8.33%) | 1 (6.67%) |  |
| Glucose (mmol/L) | 6.69 (5.90, 8.59) | 8.17 (6.96, 11.54) | **<.001** | 7.04 (6.33, 8.21) | 7.03 (6.27, 9.31) | 0.965 |
| Albumin (g/L) | 32.90 (24.90, 40.70) | 34.90 (28.00, 42.30) | 0.362 | 35.05 (26.35, 40.62) | 31.90 (23.18, 39.80) | 0.598 |
| Scr (μmol/L) | 69.00 (56.90, 94.70) | 75.00 (56.00, 146.30) | 0.346 | 77.15 (55.35, 123.40) | 75.00 (68.50, 110.25) | 0.740 |
| LDH (U/L) | 215.00 (173.00, 253.00) | 230.00 (194.00, 314.00) | 0.292 | 240.50 (193.00, 295.50) | 196.00 (173.50, 221.50) | 0.063 |
| CRP (mg/L) | 32.75 (5.17, 82.98) | 70.01 (12.87, 179.15) | **0.035** | 25.92 (4.75, 103.06) | 41.61 (20.59, 98.09) | 0.273 |
| D-dimer (mg/L) |  |  | 0.393 |  |  | 1.000 |
| ≤ 0.5 | 10 (17.54%) | 10 (12.35%) |  | 2 (8.33%) | 1 (6.67%) |  |
| > 0.5 | 47 (82.46%) | 71 (87.65%) |  | 22 (91.67%) | 14 (93.33%) |  |

Table S6 Logistic regression analysis for the association between NLR, SII, and intestinal necrosis in acute intestinal ischemia patients after PSM.

| Variable | OR (95%CI) | *P* |
| --- | --- | --- |
| NLR |  |  |
| < 8.85 | 1.000 (Reference) |  |
| ≥ 8.85 | 4.444 (1.076 - 18.355) | **0.039** |
| SII (*10^9^) |  |  |
| < 1492.11 | 1.000 (Reference) |  |
| ≥ 1492.11 | 6.679 (1.576 - 28.293) | **0.010** |
